# Undershoot Recovery in Polystyrene Melts: Effects of Annealing on Repeated Shear Startup

**DOI:** 10.1021/acs.jpcb.5c05918

**Published:** 2025-12-29

**Authors:** Benke Li, Dimitris Vlassopoulos

**Affiliations:** † 124215 Institute of Electronic Structure and Laser, FORTH, Heraklion 70013, Greece; ‡ Department of Chemical and Biomolecular Engineering, 5972 University of Delaware, Newark, Delaware 19716, United States; § Department of Materials Science and Engineering, University of Crete, Heraklion 70013, Greece

## Abstract

Entangled polymer melts undergo a transient stress undershoot, following the well-known overshoot, during shear startup, as established with several experiments using primarily nearly monodisperse linear polystyrenes (PS) and their mutual blends of different molar mass. While the microscopic origin of the undershoot remains debated, growing evidence supports a connection to chain tumbling, as proposed by [ CostanzoS.,  Macromolecules 2016, 49­(10), 3925–3935.] through their tumbling-enabling Ianniruberto–Marrucci (IM) model and further supported by simulations and other modeling approaches. The current view is that undershoots reflect the cyclic orientation-retraction dynamics of chains with the initial chain orientation state playing a decisive role. Here, we investigate both overshoot and undershoot behavior systematically across a series of PS melts across the unentangled-entangled transition (with the average number of entanglements ranging from 1.8 to 16.7) using a modular cone-partitioned-plate (CPP) geometry, and compare the experimental data with the tumbling-enabling Ianniruberto-Marrucci (IM) model. Subsequently, repeated shear startup protocols with increasing rest (annealing) times between sequential tests were applied to probe the undershoot evolution. Our results demonstrate that undershoots re-emerge only after unexpectedly long annealing times at sufficiently large shear rates (*Wi_R_* ≥ 10), highlighting persistent structural memory effects. These findings provide new insights on how segmental reorientation affects the undershoot recovery of sheared polymer melts and contributes toward assessing and improving the constitutive description of nonlinear polymer dynamics.

## Graphical abstract

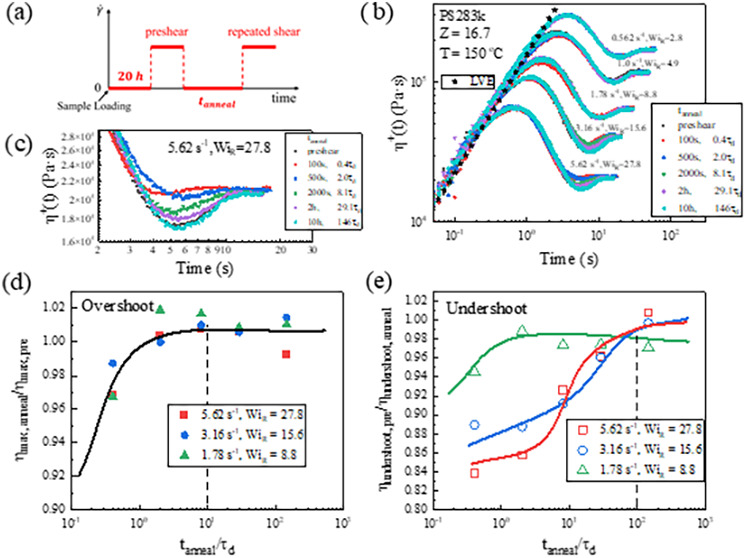

## 1 Introduction

Understanding the nonlinear shear response of polymer melts is essential for both processing and constitutive modeling.^1–3^ During shear startup at large shear rates, the transient stress response exhibits two unambiguous features: (i) the well-known overshoot, observed in experiments^4–9^ and simulations,^10–12^ and explained theoretically.^13^ (ii) a following undershoot, which was reported experimentally^8,14,15^ with monodisperse polystyrene (PS) melts for Rouse Weissenberg number *Wi*_*R*_ ≥ 1, here *Wi*_*R*_ is the product of shear rate γ̇ and the melt’s Rouse time τ_*R*_. Recent experimental work by Parisi et al.,^16^ reported that undershoots can be largely suppressed in binary blends when both components are entangled, suggesting a sensitive dependence on composition and the number of entanglements per chain. Unentangled polymers also exhibit a weak undershoot.^17^

Despite the solid experimental evidence for undershoots, their physical origin remains debated. The classical tube-based models like the GLaMM model^18^ and the MLD framework^19–21^ do not predict any undershoot reliably, implying that additional physics is missing. One proposed mechanism is chain tumbling^15,22,23^ under strong shear. It was first observed in simulations,^15,23–26^ that consistently showed cyclic rotation and retraction of polymeric chains at high Weissenberg numbers, and naturally produced undershoot-like signatures of the transient stress. Motivated by this, Costanzo and co-workers^8^ incorporated rotational tumbling modes into the tube-based integral constitutive model, which we call Ianniruberto–Marrucci (IM) framework (“tumbling-enabling IM”), and it predicts undershoot. Wagner and co-workers^27^ took an alternative route with the rotation-zero-stretch (RZS) model. While describing experimental overshoots and steady viscosities very well, RZS predicts weak undershoots. An alternative explanation by Xie and Schweizer^28^ attributes undershoots to a nonlinear coupling between chain stretch and orientational stress, described as a deformation-induced “grip force” concept originally proposed by Wang et al.,^29^ in which the undershoot appeared by delaying the onset of convective constraint release (CCR). This viewpoint was challenged in a recent study comparing different modeling approaches for transient nonlinear shear rheology.^20^

Despite the above developments and the unsettled theoretical issues, two outstanding experimental challenges are worth considering. First, how does the undershoot depend on the molar mass and whether the current predictive framework based on the tumbling idea can capture the experimental trends across the unentangled-entangled transition. Second, what is the influence of repeated shear on the undershoot. To answer the latter question, it is important first to outline the concept of repeated shear. Stratton and Butcher had proposed a protocol that comprises several consecutive shear startup tests at a fixed shear rate, separated by relaxation (recovery) at zero shear for different specified rest times, with the intention to investigate entanglement dynamics.^30^ The recovery of the transient shear stress (viscosity) overshoot to its original shape (of the first transient test) in such repeated shear experiments has been studied extensively for different entangled linear polymers, and it was revealed that a rest period of about 10 τ_*d*_ is required for complete recovery, where τ_*d*_ is the terminal relaxation time.^30–33^ The generality of the phenomenon was confirmed by investigations on branched polymers.^34–36^ However, its molecular origin is not fully understood yet.

Originally, overshoot recovery was attributed to shear-induced reentanglement, following the disentanglement that marked the initial overshoot.^31,32^ However, based on the Doi–Edwards theory, the convective constraint release (CCR) concept and molecular simulations, the effect of shear (on the initial overshoot) has been understood as a reflection of both segmental orientation and disentanglement.^25,37,38^ Using the integral form of the Doi–Edwards constitutive equation,^3^ Ianniruberto and Marrucci have shown that entanglement recovery alone cannot fully account for the experimental observations, and instead proposed that overshoot recovery is primarily governed by chain reorientation,^39^ a view also supported by Masubuchi et al.^40^ using multichain slip-link simulations. This view has been corroborated recently by Olmsted and co-workers, who performed careful molecular simulations and constitutive modeling^41–43^ and demonstrated that entanglements recover on the Rouse time scale. The emerging picture is that polymers, both linear and branched alike, recover their transient shear stress overshoot at long times. The extremely slow structural recovery reflects orientational and topological aspects (segmental orientation, reentanglement, and branch point hopping for branched polymers). We note that, unlike elongation, the chains hardly undergo irreversible scission in simple shear.^44,45^ In contrast to the overshoot, the recovery of an undershoot under repeated shearing is largely unexplored.

In this work, we address the behavior of the transient shear stress undershoot under repeated shearing for different linear polymers. To this end, we first determined the linear viscoelastic spectra. Then, we used state-of-the-art experiments, i.e., the modular cone-partitioned-plate (CPP) geometry,^22,46–48^ to probe the transient shear signal of a set of nearly monodisperse PS melts with the number of entanglements ranging from *Z* = 1.8 to 16.7. The tumbling-enabling IM model (see the Appendix for the brief introduction)^8^ was employed to assess the extent and limits of current constitutive descriptions across entanglement densities. A repeated shear startup protocol with controlled rest times (annealing) interrogated the structural memory and revealed a pronounced shear-rate and time dependence of the undershoot recovery.

## 2 Materials and Experiments

### 2.1 Materials and Sample Preparation

Six atactic linear polystyrene (PS) samples were investigated. They had weight-average molar mass of *M*_w_ = 30.3, 45, 76.5, 90, 202.1, and 283.3 kg/mol, polydispersity (PD) of 1.03, 1.04, 1.04, 1.04, 1.03, and 1.02, respectively, and were coded as PS30k, PS45k, PS76k, PS90k, PS202k, and PS283k, respectively. PS45k and PS76k were obtained from Polymer Source (Canada), while PS30k, PS90k, PS202k, and PS283k were obtained from Agilent Technologies. The entanglement molar mass was taken as 17 kg/mol.^49^ Their glass transition temperatures were measured with differential scanning calorimetry (DSC) and were found to be *T*_g_ = 100 ± 0.5 °C. Figure 1 shows the linear viscoelastic (LVE) spectra of interest in this study encompassing the rubbery plateau and terminal regimes. The frequency-dependent storage and loss moduli of these polymers were measured with Small Amplitude Oscillatory Shear (SAOS) tests over the temperature range from 100 to 180 °C with stainless steel parallel-plate (PP) geometry of radius 4 mm and sample thickness between 1.6 and 2.2 mm. Time–temperature superposition (TTS) was applied to construct master curves at a reference temperature of *T*_ref_ = 150 °C (Figure 1a). To this end, the data were shifted, and the horizontal shift factor *a*_*T*_ and vertical shift factor *b*_*T*_ are shown in Figure 1b as a function of temperature. The *a*_*T*_ data were well-fitted by the Williams–Landel–Ferry (WLF) equation^50^ (see solid lines of Figure 1b), $\log a_{T}=-\frac{C_{1}\left(T-T_{\mathrm{ref}}\right)}{C_{2}+\left(T-T_{\mathrm{ref}}\right)}$ , where *T*_ref_ = 150 °C. The *b*_*T*_ data are described by the equation (solid line in the inset of Figure 1b) $b_{T}=\frac{\rho_{\mathrm{ref}}\left(T_{\mathrm{ref}}+273.15\right)}{\rho \left(T+273.15\right)}$ , where the temperature-dependent density is ρ­(*T*) = 1.2503–6.05 × 10^–4^(273.15 + *T*).^51^ The values of *C*_1_ and *C*_2_ at the reference temperature of 150 °C and *T*_g_ were listed in Table 1, where $C_{1,T_{\mathrm{ref}}=T_{g}}=\frac{C_{1}C_{2}}{C_{2}+T_{g}-T_{\mathrm{ref}}}$ and *C*_2,*T*_ref_=*T*_g__ = *C*_2_ + *T*_g_–*T*_ref_. The data are satisfactory and consistent with earlier analysis with polystyrenes of similar molar mass.^9^ Further evidence for the quality of the data comes from a comparison with modeling. The master curves (*T*_ref_ = 150 °C) were fitted with the state-of-the-art tube model, known as Likhtman-McLeish (LM) model,^52^ for linear entangled polymers. This model makes use of three independent parameters: the number of entanglements *Z*, the rubbery plateau modulus *G*_*e*_ = ρ*RT*/*M*_*e*_= 0.209 MPa and, the Rouse time of an entanglement segment τ_*e*_. The fitting results are shown in Figure 1c (solid lines), and the fit parameters are listed in Table 1, with the other parameters calculated as plateau modulus *G*_*N*_^0^ = 4/5 *G*_*e*_, Rouse time τ_*R*_ = *Z* ^2^τ_*e*_, terminal time τ_*d*_ = 3*Z* ^3^τ_*e*_, respectively^52^ (the nearly same relaxation times per mode reflect that fact that for the different combinations of molar mass and temperature investigated, the terminal relaxation times were very close). An additional parameter is the CCR parameter *c*_ν_.^5,18,53^ Figure 1c confirms that the LM model^52^ describes the linear viscoelastic data very well.

**Figure 1:**
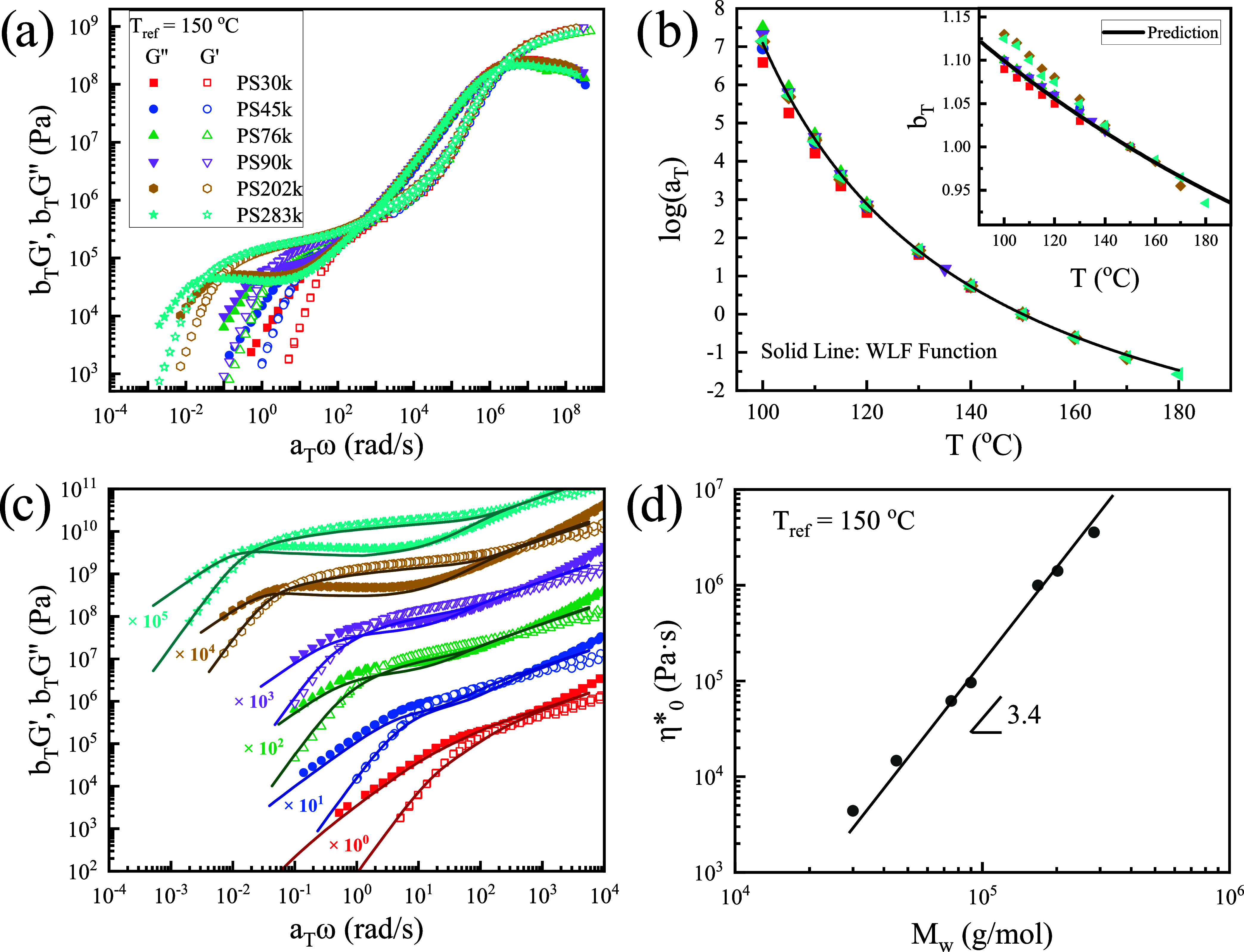
Linear viscoelastic (LVE) characterization of PS melts with a reference temperature of 150 °C. (a) Master curves obtained by TTS using both horizontal and vertical shift factors (*a*_*T*_, *b*_*T*_); raw data were corrected for geometry compliance.^54,55^ (b) Horizontal shift factor log *a*_*T*_ versus temperature. Symbols are experimental data, and the solid black line shows the average WLF^50^ fit, while all fit parameters for each sample are listed in Table 1. Inset: vertical shift factor *b*_*T*_ versus temperature; the solid line is the density-based prediction $b_{T}=\frac{\rho_{\mathrm{ref}}\left(T_{\mathrm{ref}}+273.15\right)}{\rho \left(T+273.15\right)}$ . (c) TTS master curves (symbols) together with LM model^52^ fits (solid lines). A common entanglement Rouse relaxation time τ_*e*_ was used for all samples, and the CCR parameter *c*_ν_ was adjusted per sample (see Table 1). To aid visualization, each data set is vertically offset by the factor indicated (e.g., × 10^*n*^). (d) Zero-shear complex viscosity η_0_^*^ obtained from the terminal region of the master curves, plotted vs molar mass *M*_*w*_. The line indicates power-law scaling η_0_^*^∼*M*_*w*_^3.4^.

**Table 1:** Material Properties, WLF^50^ and LM^52^ Fitting Parameters

	PS30k	PS45k	PS76k	PS90k	PS202k	PS283k
*M*_*w*_ (kg/mol)	30.3	45	76.5	90	202.1	283.3
PD = *M_w_*/*M_n_*	1.03	1.04	1.04	1.04	1.03	1.02
*T*_g_ (°C)	100.1	100.2	100.5	100.4	100.5	100.3
*Z*	1.8	2.7	4.5	5.3	11.9	16.7
*C*_1,*T*_ref_ = 150 °C_	5.44	5.75	5.45	5.63	5.74	5.98
*C*_2,*T*_ref_ = 150 °C_	91.35	91.35	86.35	88.57	90.42	92.18
*C*_1,*T*_ref_=*T*_g__	11.98	12.63	12.78	12.80	12.69	12.98
*C*_1,*T*_ref_=*T*_g__	41.45	41.55	36.85	38.97	40.92	42.48
*c*_υ_	0.0	0.003	0.03	0.08	0.1	0.3
τ_*e*,150 °C_ (ms)	17.7^b^	17.7	17.7	17.7	17.7	17.7
τ_*R*,150 °C_ (s)	0.057^b^	0.124	0.358	0.497	2.51	4.94
τ_*d*,150 °C_ (s)	0.31^b^	0.99	4.84	7.91	89.5	247.3
*T*_test_ (°C)	120	125	130	135	150	150
*a*_*T*_ at *T*_test_ ^a^	460	147	46.0	15.2	1.0	1.0

a The relaxation times τ_*e*_, τ_*R*_, and τ_*d*_ under test (shear startup) temperatures were calculated by *a*_*T*_ at test temperatures multiply those times under 150 °C, respectively.

b PS30k lies on the unentangled-entangled crossover; for consistency, it was also fitted with the LM model using the same τ_*e*_ and relations τ_*R*_ = Z_*e*_^2^ and τ_*d*_ = 3Z_*e*_^3^.

**Table 2:** Maxwell Model Fits Parameters of Linear Viscoelasticity (LVE)

	*g*(Pa)					
τ_*i*_(s)	PS30k 120 °C	PS45k 125 °C	PS76k 130 °C	PS90k 135 °C	PS202k 150 °C	PS283k 150 °C
1.00 × 10^–4^	1.96 × 10^8^	2.92 × 10^8^	1.37 × 10^8^	5.05 × 10^7^	5.29 × 10^6^	5.12 × 10^6^
2.51 × 10^–4^	1.01 × 10^8^	-	-	-	-	-
6.31 × 10^–4^	9.26 × 10^7^	1.25 × 10^6^	-	-	-	-
1.59 × 10^–3^	9.59 × 10^6^	4.85 × 10^6^	3.71 × 10^5^	-	6.44 × 10^4^	3.52 × 10^4^
3.98 × 10^–3^	3.93 × 10^6^	-	1.08 × 10^6^	4.50 × 10^5^	1.96 × 10^5^	1.87 × 10^5^
1.00 × 10^–2^	1.02 × 10^6^	9.88 × 10^5^	-	3.02 × 10^5^	-	-
2.51 × 10^–2^	2.93 × 10^5^	7.72 × 10^3^	3.82 × 10^5^	6.67 × 10^4^	5.54 × 10^4^	4.66 × 10^4^
6.31 × 10^–2^	6.45 × 10^5^	2.58 × 10^5^	9.32 × 10^4^	1.27 × 10^5^	1.58 × 10^4^	1.47 × 10^4^
1.58 × 10^–1^	1.49 × 10^5^	2.33 × 10^5^	1.00 × 10^5^	4.27 × 10^4^	2.80 × 10^4^	2.29 × 10^4^
3.98 × 10^–1^	1.76 × 10^5^	5.69 × 10^4^	7.91 × 10^4^	5.40 × 10^4^	2.18 × 10^4^	1.68 × 10^4^
1.00	1.73 × 10^5^	1.08 × 10^5^	2.53 × 10^4^	2.64 × 10^4^	2.74 × 10^4^	2.54 × 10^4^
2.51	-	2.59 × 10^4^	6.34 × 10^4^	5.25 × 10^4^	3.19 × 10^4^	1.84 × 10^4^
6.31	1.88 × 10^5^	7.54 × 10^4^	1.28 × 10^4^	2.39 × 10^4^	2.63 × 10^4^	3.67 × 10^4^
1.58 × 10^1^	1.99 × 10^4^	6.77 × 10^4^	6.58 × 10^4^	6.93 × 10^4^	5.36 × 10^4^	1.15 × 10^4^
3.98 × 10^1^	-	-	3.36 × 10^4^	-	7.24 × 10^3^	5.43 × 10^4^
1.00 × 10^2^	5.26 × 10^2^	1.68 × 10^3^	1.36 × 10^3^	-	-	9.14 × 10^3^

### 2.2 Melt Rheology

To perform rheological measurements, the samples were shaped into discotic specimens by using vacuum compression molding. Rheological measurements were performed on ARES strain-controlled rotational rheometers (TA) equipped with force rebalance transducers (FRT). Nitrogen convection ovens were used for temperature control and creating an inert atmosphere. The linear viscoelastic tests were measured with an 8 mm diameter parallel-plate geometry. For all reported data, the transducer compliance effects were corrected following the procedure reported in literature.^54,55^ The startup nonlinear shear tests were performed with stainless steel CPP geometries.^22,46–48^ The bottom fixture was a cone with a diameter of 25 mm, cone angle of 0.1 rad, and a truncation of 53 μm. The upper fixture consisted of an inner measuring plate and an outer inert (nonmeasuring) ring attached to the head of the transducer. The radius of the inner plate used was *R*_inner_ = 2 mm.

## 3 Results and Discussion

### 3.1 Linear Viscoelasticity

Figure 1 depicts the linear viscoelastic data of the PS investigated. Table 1 summarizes the material properties, WLF^50^ and LM^52^ fitting parameters. Figure 1a shows the master curves constructed by time–temperature superposition (TTS) at the reference temperature of 150 °C. The master curves of different molar masses collapse in the high-frequency regime, and this observation is consistent with the DSC data summarized in Table 1, which confirms that all samples have nearly identical *T*_g_.

Figure 1b shows the temperature dependence of horizontal shift factor *a*_*T*_ and vertical shift factor *b*_*T*_. The experimental data (symbols) are well described by the WLF equation.^50^ For clarity, one average WLF fit across all PS melts is shown in the figure, while the individual fit parameters *C*_1_ and *C*_2_ for each sample are listed separately in Table 1. Two reference temperatures were chosen: *T*_ref_ = 150 °C and *T*_ref_ = *T*_g_. For *T*_ref_ = 150 °C, the fitted constants are *C*_1_ = 5.4∼6.0 and *C*_2_ = 86∼92 K. These values are typical for WLF fits referenced significantly above the glass transition. For *T*_ref_ = *T*_g_, the fitted constants are *C*_1_ = 12∼13 and *C*_2_ = 37∼43 K. These values are close to the “universal” WLF constants originally reported by Williams, Landel, and Ferry (*C*_1_ = 17.44, *C*_2_ = 51.6).^50^ The weak scattering of the horizontal shift factors at low temperatures reflects subtle differences in segmental mobility and free-volume characteristics near the *T*_g_.

In Figure 1c, the master curves were fitted with the Likhtman–McLeish (LM) model,^52^ shown as solid lines, where the fitting parameters were summarized in Table 1. An entanglement Rouse relaxation time of τ_*e*_ = 17.7 ms was used for all samples. The main adjustable parameter in the fitting is *c*_ν_, the coefficient of convective constraint release (CCR). As summarized in Table 1, *c*_ν_ increases systematically with molar mass, from 0.003 for PS45k to 0.3 for PS283k. In most tube models, *c*_ν_ is typically fixed around 0.1,^53^ reflecting a nearly universal value for linear viscoelastic fits. Although *c*_ν_ is often referred to as the “CCR parameter”, under linear viscoelastic (SAOS) conditions, it represents thermal constraint release (CR) rather than convective constraint release, since chain configurations are only weakly perturbed from equilibrium. Moreover, because *c*_ν_ is related to the number of retraction events required for one CR hop, its variation with molar mass may reflect differences in the chain-end density and constraint-release efficiency among the samples.

Finally, Figure 1d shows the zero-shear complex viscosity η_0_^*^ as a function of molar mass *M*_*w*_ at *T*_ref_ = 150 °C. The data are consistent with the well-known scaling η_0_ ∼ *M*_*w*_^3.4^.

### 3.2. Shear Startup

Figure 2 shows the shear startup response of the PS melts with molar masses ranging from virtually unentangled PS30k (*Z* = 1.8)^17^ to highly entangled PS283k (*Z* = 16.7), at imposed shear rates that range from 0.01 to 31.6 s^–1^. The corresponding *Wi*_*d*_ and *Wi*_*R*_ are also shown in the figures. Specifically, the shear stress growth coefficient (or transient viscosity) is plotted as a function of time for different shear rates. It can be observed that at sufficiently high shear rates (*Wi_R_* > 1), all samples exhibit a pronounced overshoot in viscosity. The predictions of the tumbling-enabling IM model^8^ are shown as solid lines (β = 0.5) and dashed lines (β = 2.0), where β is an unknown parameter used to tune the tube loss rate associated with the CCR mechanism under strong flow (also see Appendix).^8^ The Rouse relaxation times were obtained from LM model fitting (see Table 1), and the multimode relaxation spectra were obtained from generalized Maxwell model fits (see Table 2). For samples with a large number of entanglements (PS202k, *Z* = 11.9; PS283k, *Z* = 16.7), the tumbling-enabling IM model provides a good description of both the overshoot and undershoot. However, as the molar mass decreases, particularly near the crossover regime between unentangled and weakly entangled, the discrepancies between the model and experiment become more pronounced. The most notable deviations are the fact that the IM model systematically overpredicts the magnitude of the overshoot and the respective strain at the overshoot. Adjusting the model parameter β from 0.5 to 2.0 does not significantly improve the predictions, indicating the limitations of capturing the transient shear behavior in the low-entanglement regime. It should be noted that the GLaMM model^18^ also exhibits significant deviations from the experimental data in the crossover regime (*Z* = 2–5). In this work, we focus on the use of the tumbling-enabling IM model.

**Figure 2:**
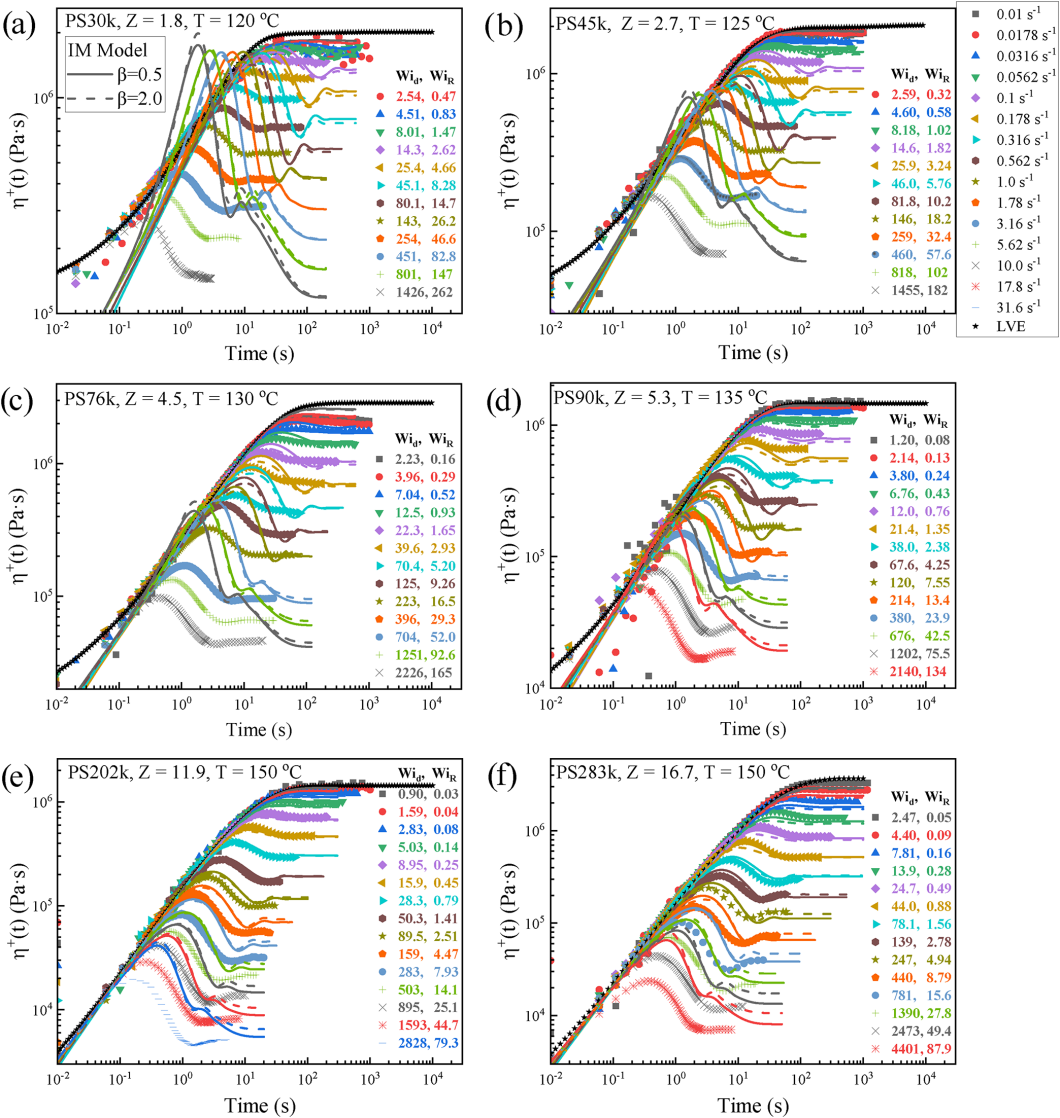
Nonlinear startup shear of polystyrene melts with different molar masses and entanglement numbers. (a−f) PS30k (*Z* = 1.8, *T* = 120 °C), PS45k (*Z* = 2.7, *T* = 125 °C), PS76k (*Z* = 4.5, *T* = 130 °C), PS90k (*Z* = 5.3, *T* = 135 °C), PS202k (*Z* = 11.9, *T* = 150 °C), and PS283k (*Z* = 16.7, *T* = 150 °C). Symbols denote experimental data, solid lines represent tumbling-enabling IM model^8^ predictions with β = 0.5, and dashed lines represent that predictions with β = 2.0. All samples share the same shear-rate legend shown in the upper-right corner, while the *Wi*_*d*_ and *Wi*_*R*_ legends are indicated within each subfigure. The tumbling-enabling IM model was applied in multimode form using relaxation spectra obtained from Maxwell fits (Table 2).

Figure 3a–c plot the steady-state viscosity normalized by its zero-shear limiting value η_steady_/η_0_, the maximum stress at the overshoot σ_max_/*G*_*e*_ and the overshoot viscosity ratio η_max_/η_steady_ as a function of the terminal Weissenberg number *Wi*_*m*_ = γ̇τ_*d*_. The scaling exponents satisfy the relationship: slope­(σ_max_) = 1 – slope­(η_max_/η_steady_) – slope­(η_steady_). The steady viscosity of higher entangled samples (PS202k, *Z* = 11.9; PS283k, *Z* = 16.7) exhibits power-law slopes of −0.90 and −0.91, consistent with literature reports^4,5,8,9^ and with the IM model predictions as shown in Figure 3a. As the entanglements number *Z* decreases, the slope gradually approaches −0.50, which is the scaling for unentangled melts.^17^ This slope change is captured well by the IM model, as shown in Figure 3a. The behavior of unentangled melts has been extensively explored by experiments,^17,56,57^ simulations,^17,24,25,58–61^ and modeling.^9,17,27,62–67^ Experimentally, Stratton^56^ first reported a slope of – 0.5 for unentangled PS using capillary rheometry, that was later confirmed by Santangelo and Roland^57^ with startup shear using a rotational rheometry. NEMD simulations^17,24,25,58–61^ have successfully reproduced these findings. Constitutive modeling efforts have followed three main directions: monomeric friction-reduction concept by Ianniruberto and Marrucci^62,63^ and subsequently refined by Watanabe et al.^64^ to include the Brownian force; integral constitutive models with Rouse-type relaxation developed by Wagner and co-workers;^27,65,66^ the tension-blob/shear-slit framework, originated by Colby and co-workers,^67^ later refined by Parisi et al.^9^ and Dalne et al.^17^ to include confinement, transient overshoots, and advection. Despite these advances, the transition regime between weakly and strongly entangled melts remains challenging, likely due to tube-end effects in slightly entangled systems.

**Figure 3:**
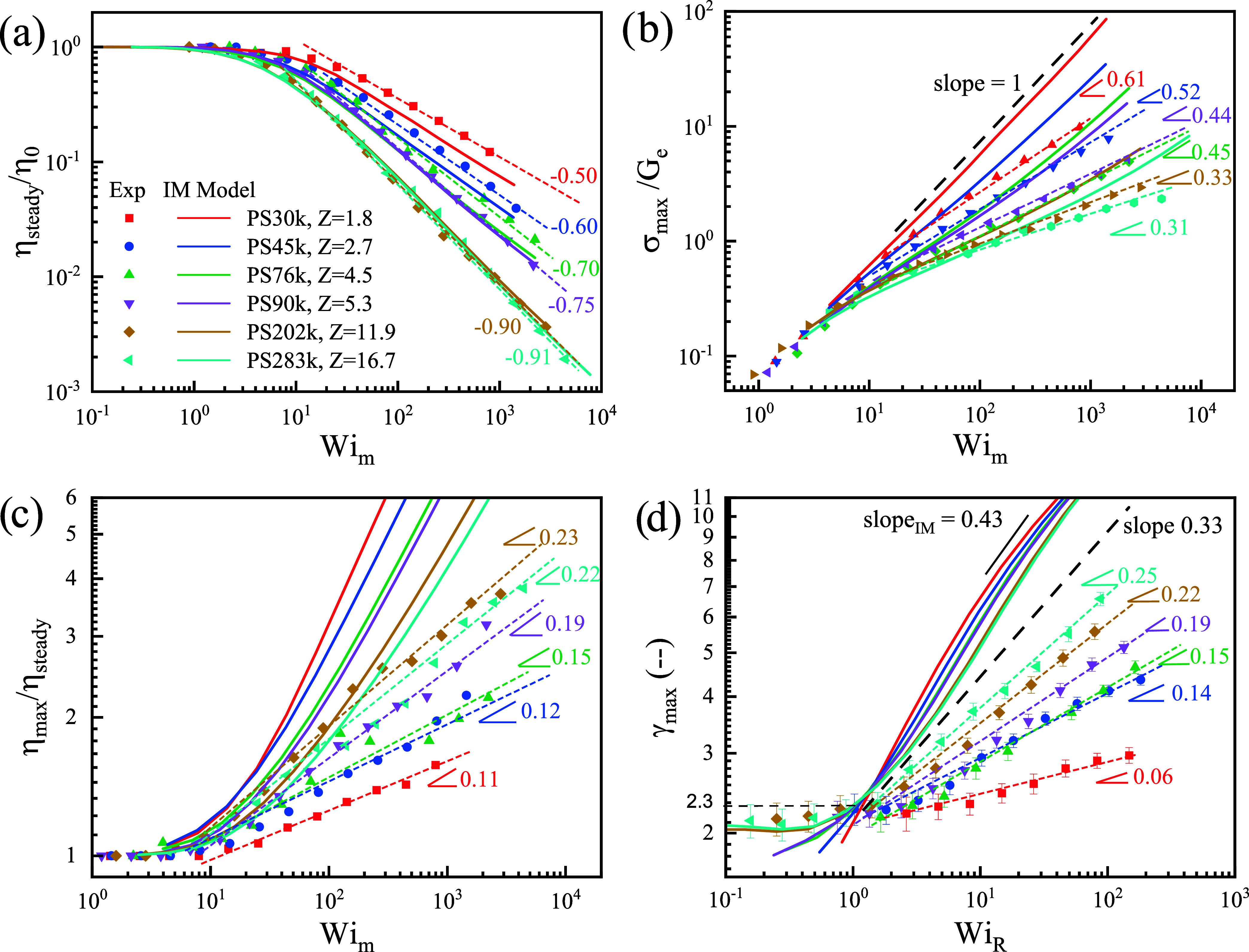
Comparison of steady-state viscosity and overshoot behavior of experimental results with tumbling-enabling IM model predictions using the parameter of β = 0.5. (a) η_steady_/η_0_ versus *Wi*_*m*_. (b) σ_max_/*G*_*e*_ versus *Wi*_*m*_. (c) η_max_/η_steady_ versus *Wi*_*m*_. (d) γ_overshoot_ versus *Wi*_*R*_. Here, the terminal Weissenberg number *Wi*_*m*_ = γ̇τ_*d*_ and the Rouse Weissenberg number *Wi*_*R*_ = γ̇τ_*R*_.

Figure 3b plots the maximum stress, σ_max_/*G*_*e*_ versus *Wi*_*m*_. For highly entangled samples (*Z* = 11.9, 16.7), the slope of σ_max_/*G*_*e*_ is 0.33 and 0.31, consistent with experiments^4–9^ and simulations,^10,11^ where an exponent of 0.30 ± 0.03 has been reported for *Wi*_*R*_ > 1. This exponent deviates from the GLaMM tube model prediction,^18^ but agrees with slip-link models^68^ and the framework of Xie and Schweizer.^13,28^ The tumbling-enabling IM model,^8^ as shown in Figure 3b, captures the behavior at low shear rates but deviates at high rates. For weakly entangled melts (*Z* = 1.8), the slope increases to 0.61, further deviating from the IM model. For unentangled polymers, recent developments such as the shear-slit model^9,17^ provide good agreement with experimental trends.

Figure 3c plots the overshoot viscosity ratio η_max_/η_steady_ versus *Wi*_*m*_, with scaling exponents that decrease as Z decreases (e.g., from 0.23 at *Z* = 16.7 to 0.11 at *Z* = 1.8). In general, the IM model could only capture the behavior of the samples with high *Z* and under low shear rates.

Figure 3d plots the strain γ_max_ at the viscosity overshoot of the shear startup curves. The overshoot strain γ_max_ remains close to the Doi–Edwards prediction of 2.3 (without the independent alignment approximation) for *Wi*_*R*_ < 1, where tube orientation dominates without chain stretch. For *Wi*_*R*_ > 1, γ_max_ increases with *Z*, approaching a slope of about 0.33 at large *Z*. This scaling has been confirmed in the literature: Wang et al.^69^ reported exponents near 0.33 for highly entangled SBR (*Z* > 24), and Costanzo et al.^22^ obtained similar values for PS melts and solutions (*Z* = 10 ∼ 14). Slightly smaller slopes were observed for PS compared to SBR at comparable Z. For unentangled melts, the slope decreases further with molar mass.^17^ The GLaMM model^18^ predicts a much steeper slope of 1, while the tumbling-enabling IM model^8^ yields 0.43 (see Figure 3d). Schweizer and Xie^13^ proposed a theory based on the grip-force concept of Wang et al.,^29^ that yielded a slope of 0.33, but the exact molecular origin of this mechanism remains debatable.^20^

Figures 4a,b summarize the undershoot behavior and tumbling-enabling IM model^8^ predictions of PS melts during startup shear. The transient viscosity undershoots phenomena of PS melts emerge once *Wi*_*R*_ > 1 during the startup behavior, consistent with previous work.^8,14^

**Figure 4:**
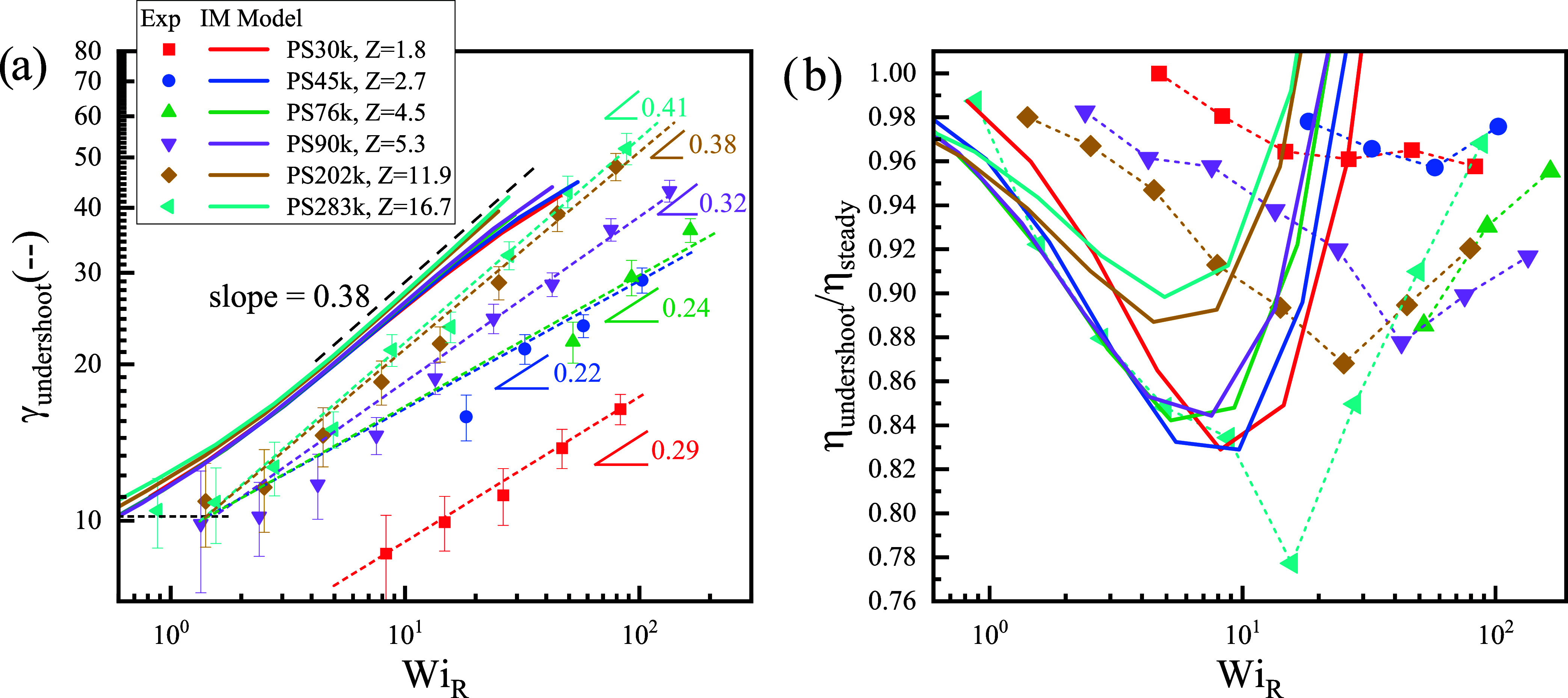
Undershoot behavior of PS melts in startup shear using CPP geometry: (a) peak strain γ_undershoot_∼*Wi*_*R*_; (b) ratio of undershoot peak viscosity to the steady-state viscosity η_undershoot_/η_steady_ versus *Wi*_*R*_. Symbols denote experimental data, while solid lines represent predictions of the tumbling-enabling IM model^8^ with parameter β = 0.5.

In Figure 4a, the undershoot strain γ_undershoot_ remains close to 10 when *Wi*_*R*_ ≈ 1, and then increases with increasing *Wi*_*R*_. The slope depends on the molar mass: for highly entangled polymers PS202k (*Z* = 11.9) and PS283k (*Z* = 16.7), the slopes are 0.38 and 0.41, respectively, which are in good agreement with the tumbling-enabling IM model^8^ prediction (slope = 0.38). However, as the molar mass decreases, the slope is reduced: for *Z* = 5.3, 4.5, and 2.7, the slopes are 0.32, 0.24, and 0.22, respectively, whereas the IM model predicts nearly constant scaling independent of entanglement density. Figure 4b shows the undershoot strength η_undershoot_/η_steady_ (the ratio of the undershoot peak viscosity to steady-state viscosity). For small entanglements (*Z* = 1.8 and 2.7), the ratio η_undershoot_/η_steady_ remains virtually constant, close to 0.97 across all *Wi*_*R*_. In contrast, with increasing *Z*, the ratio becomes more pronounced, following a U-shaped trend with *Wi*_*R*_: it first decreases and then recovers, with the minimum reaching 0.78 for PS283k (*Z* = 16.7). The turning point of this U-shape occurs in the range *Wi*_*R*_ = 10–40. The IM model also predicts a U-shaped response for all molar masses with peaks at somewhat lower rates (range *Wi*_*R*_ = 5–10). Unlike the experiments, however, the model suggests a deeper U-shape for low molar mass systems and a shallower one for highly entangled polymers.

### 3.3 Repeated Shear Startup Protocol

Figure 5a illustrates the experimental protocol of repeated shear startup, which comprises a first annealing step of 20 h after loading samples, a preshear step, a second annealing for a controlled duration (rest time), and a subsequent repeated shear at the same shear rate. Figure 5b presents the repeated shear results of PS283k obtained by using this protocol. The transient viscosity responses show the typical overshoot-undershoot sequence, with both features gradually recovering as the annealing time increases. A magnified view of the undershoot region for the highest shear rate (γ̇ = 5.62 s^–1^, *Wi*_*R*_ = 27.8) from Figure 5b is provided in Figure 5c. Figures 5d,e quantify the recovery behavior of the overshoot and undershoot peaks versus normalized annealing time *t*_anneal_/τ_*d*_, respectively. As shown in Figure 5d, the overshoot recovery is nearly independent of shear rate: data corresponding to different *Wi*_*R*_ values collapse onto a single master curve, recovering after times on the order of 10 τ_*d*_. This trend is consistent with prior experimental observations reported for entangled polymer solutions,^30,70^ entangled polymer melts,^31–33,71–73^ and even unentangled melts,^57^ where the magnitude of the stress overshoot recovers systematically with increasing rest time. As discussed in the Introduction, this behavior is commonly interpreted as a consequence of primarily chain reorientation (with the slow disentanglement–reentanglement process not being excluded but playing a secondary role). On the other hand, Figure 5e demonstrates that the undershoot recovery is strongly dependent on the applied shear rate. For *Wi*_*R*_ < 10, the undershoot recovery occurs on a similar time scale as the overshoot (∼10 τ_*d*_). However, for *Wi*_*R*_ > 10 the undershoot requires much longer annealing times, on the order of 100 τ_*d*_, to fully recover.

**Figure 5:**
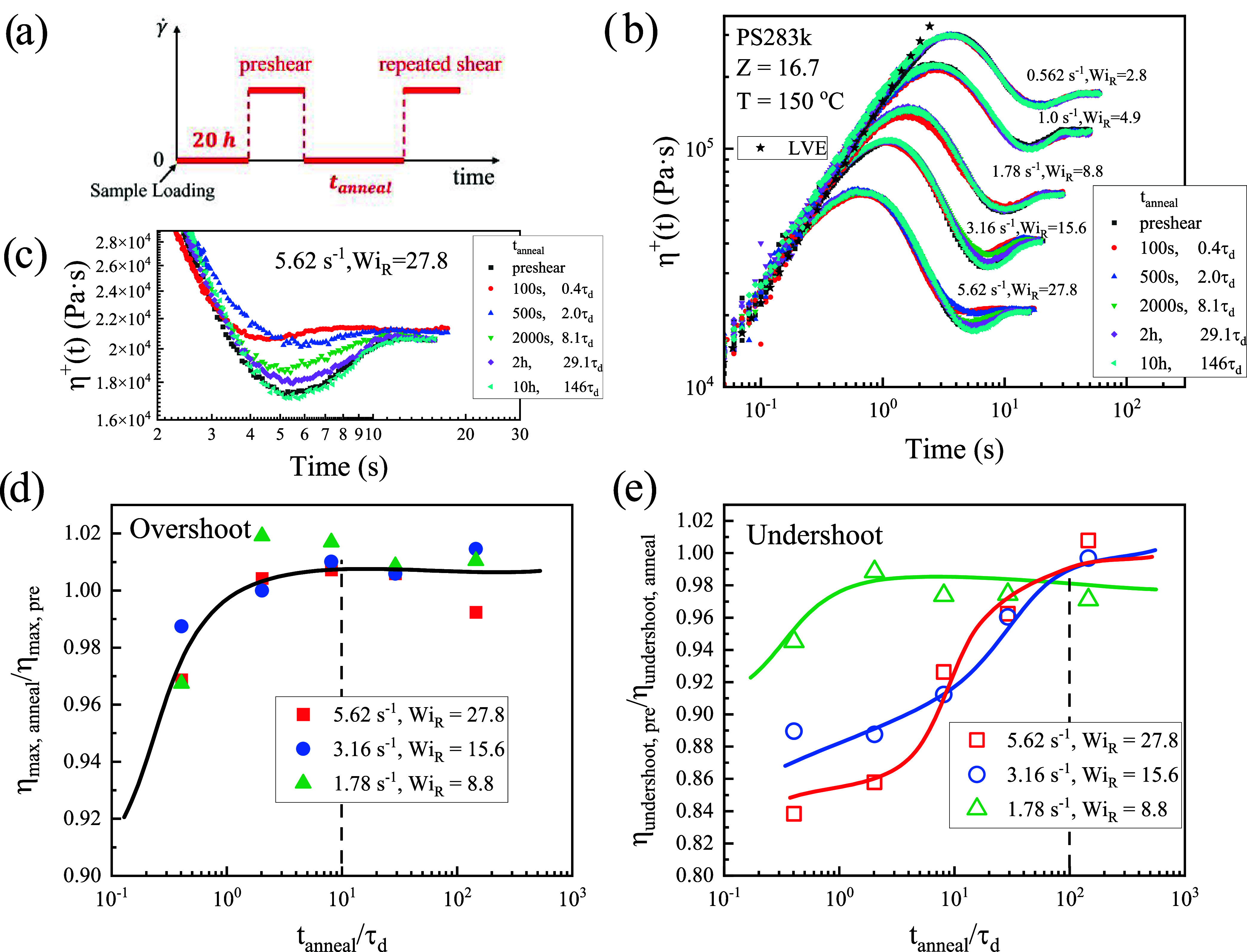
Repeated shear startup experiments of PS283k at 150 °C. (a) Schematic of the shear protocols: each run consists of a first annealing processing of 20 h after loading samples, preshear process, followed by second annealing process with annealing time varies from 100 s to 10 h, and repeated shear with the same shear rate. (b) Transient shear viscosity responses at different second annealing times. The black star symbols are the linear viscoelasticity (LVE) data obtained from the SAOS. (c) Magnified view of the undershoot region for the highest shear rate (γ̇ = 5.62 s^–1^, *Wi*_*R*_ = 27.8) from figure (b), showing details of the undershoot recovery with annealing time. (d) Recovery of overshot viscosity η_max,anneal_/η_max,pre_ and (e) recovery of undershoot viscosity η_undershoot,pre_/η_undershoot,anneal_ versus second annealing time *t*_anneal_/τ_*d*_.

This rate-dependent slowdown of undershoot recovery has not been widely reported in the literature since most previous studies focused primarily on overshoot behavior, as discussed in the Introduction. The unusually slow recovery of the undershoot at high shear rates (Figure 5d) likely points to additional structural changes induced by strong flow, possibly due to tumbling and associated density fluctuations or altered packing, that are not captured by standard tube-model frameworks. We speculate that such slow recovery may also stem from the formation of long-lived, thread-like entanglements between collapsed and extended chains under strong flow, which act as transient frictional junctions and significantly retard structural reorientation. Similar long-lived structural memory effects have been reported in confined polymer systems and entangled melts^74,75^

To assess whether the undershoot recovery is specific to highly entangled systems, we extended the repeated-startup protocol (Figure 5a) to a broader set of PS melts, ranging from a virtually unentangled PS30k (*Z* = 1.8) to weakly entangled PS45k (*Z* = 2.7), PS90k (*Z* = 5.3), and PS202k (*Z* = 11.9). The results are summarized in Figure 6. They reveal that for polymers of lower molar mass, significantly higher shear rates (*Wi_R_* ≈ 32–134) are required compared to higher molar mass (*Wi_R_* ≥ 10, Figure 5) for the recovery behavior to become apparent. Both unentangled PS30k (*Z* = 1.8) and weakly entangled PS90k (*Z* = 5.3) exhibit clear undershoot recovery under these conditions. For PS45k (*Z* = 2.7), the undershoot itself is intrinsically weak, yet careful inspection still reveals a recovery. However, despite strong experimental evidence, the molecular origin of the weak undershoot in PS45k remains unclear.

**Figure 6:**
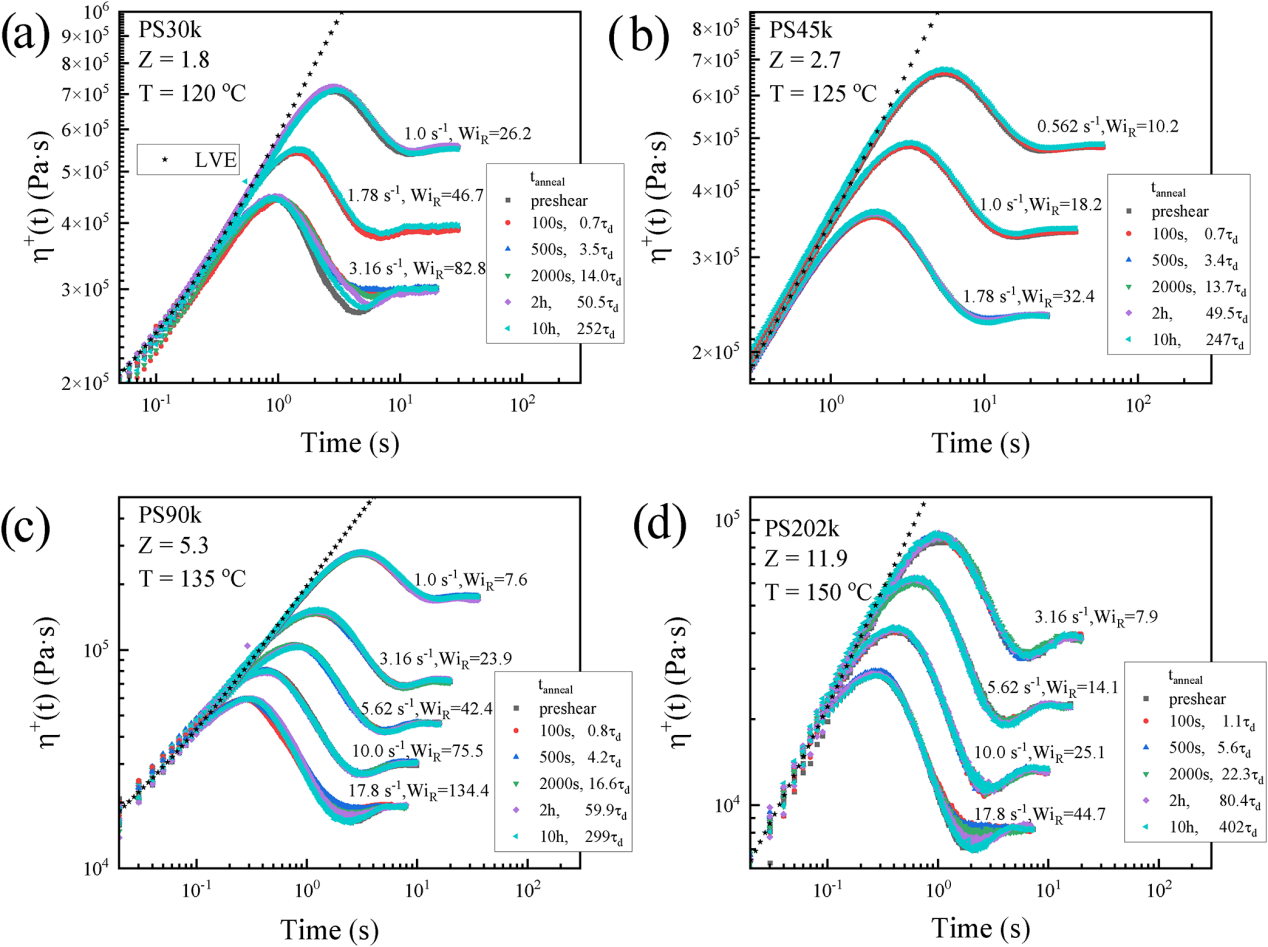
Repeated startup shear experiments of PS melts with different molar masses (entanglement numbers z): (a) PS30k (*Z* = 1.8, *T* = 120 °C), (b) PS45k (*Z* = 2.7, *T* = 125 °C), (c) PS90k (*Z* = 5.3, *T* = 135 °C), and (d) PS202k (*Z* = 11.9, *T* = 150 °C). The experimental protocol is identical to that of Figure 5, consisting of long annealing, preshear, second annealing with variable rest time, and repeated shear. Star symbols denote the linear viscoelastic (LVE) reference response.

To better visualize the recovery dynamics, Figure 7 provides magnified views of the undershoot regions for selected high shear rates, where recovery is most pronounced. These plots clearly demonstrate the progressive recovery of the undershoot magnitude with increasing annealing time, confirming that the phenomenon is a robust feature of the transient response.

**Figure 7:**
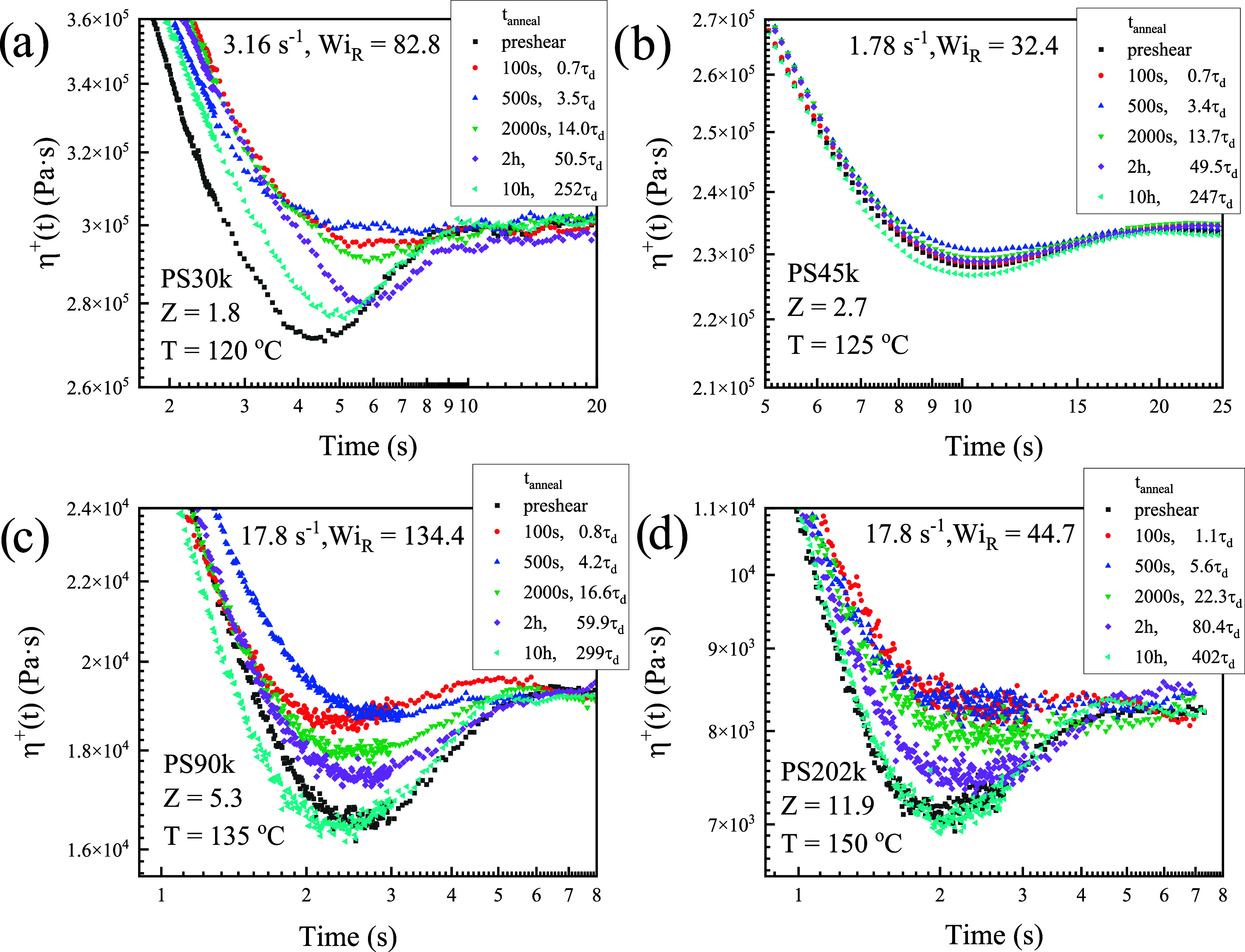
Magnified view of the undershoot recovery behavior for the PS melts of Figure 6, allowing clearer observation of the undershoot region. (a) PS30k, *Z* = 1.8 at temperature of *T* = 120 °C and shear rate of γ̇ = 3.16 s^–1^, *Wi_R_* = 82.8; (b) PS45k, *Z* = 2.7 at *T* = 125 °C, γ̇ = 1.78 s^–1^, *Wi_R_* = 32.4; (c) PS90k, *Z* = 5.3 at *T* = 135 °C, γ̇ = 17.8 s^–1^, *Wi_R_* = 134.4; and (d) PS202k, *Z* = 11.9 at *T* = 150 °C, γ̇ = 17.8 s^–1^, *Wi_R_* = 44.7.

Finally, Figure 8 consolidates the undershoot recovery data across all samples, depicting the normalized undershoot magnitude as a function of the normalized annealing time *t*_anneal_/τ_*d*_. It can be observed that across molar masses and entanglement densities, the full recovery of undershoots consistently requires long annealing times on the order of 100 τ_d_. The solid black line serves as a guide to the eye, emphasizing this common recovery time scale rather than a strong molar mass dependence.

**Figure 8:**
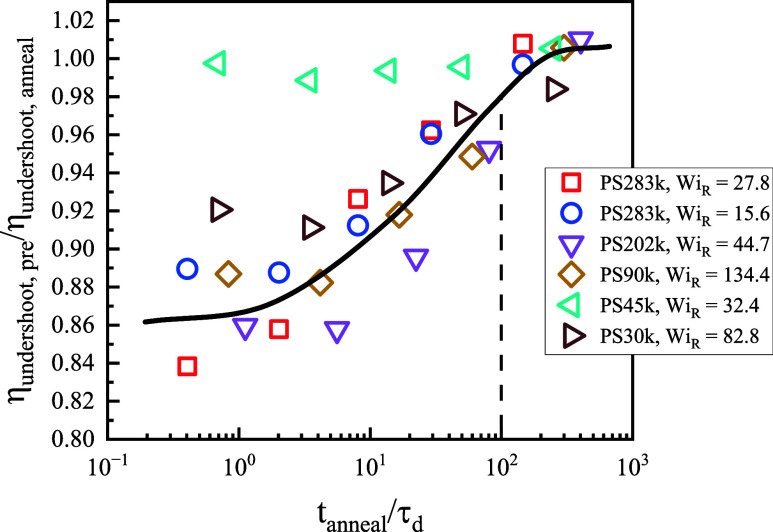
Normalized undershoot recovery of PS melts plotted as η_undershoot,pre_/η_undershoot,anneal_ versus *t*_anneal_/τ_*d*_. Symbols denote experimental data for PS283k (*Wi_R_* = 27.8, 15.6), PS202k (*Wi_R_* = 44.7), PS90k (*Wi_R_* = 134.4), PS45k (*Wi_R_* = 32.4), and PS30k (*Wi_R_* = 82.8), covering a range of entanglement densities. The solid black line serves as a guide to the eye, highlighting the general trend of recovery with increasing normalized annealing time.

## 4 Conclusions

Our systematic measurements provide new insights into the nonlinear shear response of linear monodisperse polystyrene across the unentangled-to-entangled transition. In the startup shear experiments, both viscosity overshoots and undershoots were observed at high shear rates. Comparison with the tumbling-enabling Ianniruberto–Marrucci (IM) model^8^ points to both strengths and limitations. For fully entangled melts (*Z* > 10), the model successfully reproduces the magnitude and timing of overshoots and captures the occurrence of undershoots. However, at lower entanglement densities, especially near the crossover regime (*Z* = 2–5), deviations become substantial: the model systematically overpredicts the peak stress and predicts undershoot amplitudes larger than those measured. Adjusting the model parameter β, which controls convective constraint release, did not resolve these discrepancies, indicating that additional physics is required to describe melts close to the unentangled-entangled boundary.

The repeated startup experiments further reveal critical aspects of structural memory and recovery. Whereas overshoot recovery typically requires rest times on the order of 10 τ_d_, we find that undershoot recovery exhibits a pronounced rate dependence: for *Wi_R_* < 10, undershoots recover on a comparable time scale of 10 τ_d_, but for well entangled samples at *Wi_R_* > 10 and for unentangled or weakly entangled samples at Wi_R_ in the range 32–134, full recovery requires much longer annealing times, on the order of 100 τ_d_. This behavior highlights the importance of the initial chain orientation state, which is reset only gradually upon reptation.

Overall, our findings provide systematic experimental evidence of the rate-dependent recovery of undershoots in polymer melts. The results support the idea that chain reorientation plays a decisive role in this process. Despite its shortcomings, the tumbling-enabling IM model provides a robust framework to describe the nonlinear rheology of entangled polymers, but clearly, further improvements will be necessary for enhanced accuracy. For example, it is known that in the case of rigid rods, longer rods tumble more slowly under shear flow. By analogy, it is reasonable to expect that for polymers the tumbling rate may also depend on the molar mass (or equivalently, the chain aspect ratio). Such dependence could potentially improve model predictions, especially in the weakly entangled regime. Moreover, the tumbling rate might be correlated with the degree of disentanglement. Hence, a rigorous treatment of this dependence would require additional modeling and simulation efforts in the future. More broadly, the repeated-startup protocol emerges as a sensitive tool for probing structural memory in polymer melts.

## Appendix. Brief Introduction of Tumbling-Enabling IM (Ianniruberto and Marrucci) Model

### A1 Classical CCR Model

Based on the classical constraint release rate (CCR) model proposed by Ianniruberto and Marrucci,^76^ the tube loss rate *r* was introduced to account for the CCR effect during the convective flow

1
$$\begin{array}{l} r=\kappa :S-\frac{1}{\lambda }\frac{d\lambda }{dt} \end{array}$$

here λ is the stretch ratio of the tube and evolves with time during strong flow. At steady-state shear flow *r* = **κ**:**S**. Considering the effect of tube losses, Ianniruberto and Marrucci^76^ defined an effective time τ_eff_ to replace the disengagement time τ_*d*_ in the DE model

2
$$\frac{1}{\tau_{\mathrm{eff}}}=\frac{1}{\tau_{d}}+\beta r$$

here β is an unknown coefficient, the value could be varied and adopted as 0.18,^37^ 0.5,^77^ or other values. The classical Doi-Edwards model^76^ of **S**(*t*)­

3
$$S=\int_{-\infty }^{t}\frac{dt^{\prime }}{\tau_{d}}\exp\left(-\frac{t-t^{\prime }}{\tau_{d}}\right)Q\left[E\left(t,t^{\prime }\right)\right]$$

was replaced to be

4
$$S\left(t\right)=\int_{-\infty }^{t}\left[\frac{1}{\tau_{\mathrm{eff}}\left(t^{\prime }\right)}\right]\exp\left[-\int_{t^{\prime }}^{t}\frac{dt^{\prime \prime }}{\tau_{\mathrm{eff}}\left(t^{\prime \prime }\right)}\right]Q\left[E\left(t,t^{\prime }\right)\right]dt^{\prime }$$

here tensor **Q**[**E**(*t*,*t*′)] is the Doi-Edwards nonlinear strain measure, defined by the inverse of displacement gradient tensor **E**(*t*,*t*′)

5
$$Q\equiv {\left\langle \frac{E\cdot u^{\prime }E\cdot u^{\prime }}{{\left|E\cdot u^{\prime }\right|}^{2}}\right\rangle }_{0}$$

where **u**′ is a unit vector distributed randomly over the unit sphere and ⟨ ⟩ represents an ensemble average over all **u**′. The tensor **Q** is derived using the “independent alignment approximation” (IAA).^78^

### A2 Approximation of Nonlinear Strain Measure **Q**[**E**(***t***, ***t*** ′)]

One approximation for this strain measure is^1^

6
$$Q\approx \left(\frac{5}{J-1}\right)B-\left[\frac{5}{(J-1){\left(I_{2}+13/4\right)}^{1/2}}\right]C$$

with

7
$$J\equiv I_{1}+2{\left(I_{2}+13/4\right)}^{1/2}$$

8
$$I_{1}\equiv \mathrm{Tr}\left(B\right)$$

9
$$I_{2}\equiv \mathrm{Tr}\left(C\right)$$

here *I*_1_ and *I*_2_ denote the first and second invariants of the tensor, respectively, **C** is the Cauchy tensor and **B** = **C** ^–1^ = **E·E** ^*T*^ is the Finger tensor. Another approximation of this strain measure was proposed by Milner^79^

10
$$Q\left(E\right)=\frac{B^{1/2}}{\mathrm{Tr}\left(B^{1/2}\right)}$$

which gives a much-improved prediction of the normal stress ratio in shear, namely –*N*_2_/*N*_1_ = 1/4 in the limit of small strains, as compared to Doi-Edwards value of 1/7. Here we will use Milner’s approximation.

### A3 Including the Finite Extensibility (FENE)

Furthermore, with extending to finite extensibility (FENE), λ was replaced by F­(λ),^20^ thus

11
$$\frac{d\lambda }{dt}=\lambda \kappa :S-\frac{\lambda F\left(\lambda \right)-1}{\tau_{R}}$$

12
$$F\left(\lambda \right)=\left(\frac{\lambda_{\max}^{2}-\frac{\lambda^{2}}{3}}{\lambda_{\max}^{2}-\lambda^{2}}\right)\left(\frac{\lambda_{\max}^{2}-1}{\lambda_{\max}^{2}-\frac{1}{3}}\right)$$

### A4 Including the Chain Tumbling Term during Shear Flow

The chain tumbling function under simple shear was introduced by Costanzo et al.^8^

13
$$\varphi \left(t\right)=\cos\left(\frac{Wi_{R}^{-0.2}\mathrm{\gamma ̇}t}{4}\right)\exp\left(-\frac{Wi_{R}^{-0.2}\mathrm{\gamma ̇}t}{8}\right)$$

This function was incorporated into the function

$$\frac{d\lambda }{dt}=\kappa :\overset{-}{S}\varphi \left(t\right)\lambda -\frac{F\left(\lambda \right)\lambda -1}{\tau_{R}}$$
14

Therefore, in this treatment, the tumbling function is introduced into the stretch ratio λ equation rather than directly into the orientation tensor **S**, serving as a practical compromise to capture the effect within a simplified formulation. The accuracy of this approach remains an open question.

### A5 Summarize the Multimode Tumbling-Enabling IM Model Functions^8^

15
$$S_{i}\left(t\right)=\int_{-\infty }^{t}\left[\frac{1}{\tau_{i}\left(t^{\prime }\right)}\right]\exp\left[-\int_{t^{\prime }}^{t}\frac{dt^{\prime \prime }}{\tau_{i}\left(t^{\prime \prime }\right)}\right]Q\left[E\left(t,t^{\prime }\right)\right]dt^{\prime }$$

$$\overset{-}{S}=\int_{-\infty }^{t}\left[\frac{1}{\tau_{d}\left(t^{\prime }\right)}\right]\exp\left[-\int_{t^{\prime }}^{t}\frac{dt^{\prime \prime }}{\tau_{d}\left(t^{\prime \prime }\right)}\right]Q\left[E\left(t,t^{\prime }\right)\right]dt^{\prime }$$
16

17
$$Q=\frac{B^{1/2}}{\mathrm{Tr}B^{1/2}}$$

$$\frac{d\lambda }{dt}=\varphi \left(t\right)\lambda \kappa :\overset{-}{S}-\frac{\lambda F\left(\lambda \right)-1}{\tau_{R}}$$
18

19
$$\varphi \left(t\right)=\cos\left(\frac{Wi_{R}^{-0.2}\mathrm{\gamma ̇}t}{4}\right)\exp\left(-\frac{Wi_{R}^{-0.2}\mathrm{\gamma ̇}t}{8}\right)$$

$$r=k:\overset{-}{S}-\frac{1}{\lambda }\frac{d\lambda }{dt}$$
20

21
$$\frac{1}{\tau_{i}\left(t\right)}=\frac{1}{\tau_{i,\mathrm{eq}}}+\beta r$$

22
$$\tau_{d}\left(t\right)=\frac{\sum_{i}G_{i}\tau_{i}^{2}\left(t\right)}{\sum_{i}G_{i}\tau_{i}\left(t\right)}$$

23
$$F\left(\lambda \right)=\left(\frac{\lambda_{\max}^{2}-\frac{\lambda^{2}}{3}}{\lambda_{\max}^{2}-\lambda^{2}}\right)\left(\frac{\lambda_{\max}^{2}-1}{\lambda_{\max}^{2}-\frac{1}{3}}\right)$$

24
$$\sigma =C_{Q}F\left(\lambda \right)\lambda^{2}\sum G_{i}S_{i}$$

here the subscript *i* denotes different modes, **S̅** is the average of **S**_*i*_, and *C*_*Q*_ = 6.^8^
